# MICA*049, not MICA*009, is associated with Behçet’s disease in a Chinese population

**DOI:** 10.1038/s41598-019-47289-z

**Published:** 2019-07-26

**Authors:** Weifeng Zhu, Yan Deng, Jiucun Wang, Xinjian Guo, Weifeng Ding, Jiashuo Chao, Dan Lin, Yuqin Wang, Xiaodong Zhou

**Affiliations:** 1 Department of Biochemistry and Molecular Biology, College of Basic Medical Sciences, Nanchang University, Nanchang, China; 2 Department of Internal Medicine, McGovern Medical School, University of Texas Health Science Center at Houston, Houston, Texas USA; 3 Department of Ophthalmology of Children, The Second Affiliated Hospital of Nanchang University, Nanchang, China; 4 State Key Laboratory of Genetic Engineering, Collaborative Innovation Center for Genetics and Development, School of Life Sciences, Fudan University, Shanghai, China; 5 Institute of Rheumatology, Immunology and Allergy, Fudan University, Shanghai, China; 6 Human Phenome Institute, Fudan University, Shanghai, China; 7 Department of Laboratory Medicine, Affiliated Hospital of Nantong University, Nantong, China; 8 The First Clinic Medical College, School of Medicine, Nanchang University, Nanchang, China; 9 Department of Uveitis, The Eye Hospital, Wenzhou Medical University, Wenzhou, China

**Keywords:** Immunogenetics, Vasculitis syndromes

## Abstract

Behçet’s disease (BD) is a multi-systemic inflammatory disease. Previous reports indicated that MICA*009 confers susceptibility to BD. MICA*049 differs from MICA*009:01, a major MICA*009 subtype, only at codon 335 in exon 6. However, the potential association of MICA*049 with BD  has not been addressed. In this study, we differentiated association among MICA*049, MICA*009 and HLA-B*51 with BD. A Han Chinese cohort consisting of 41 BD patients and 197 ethnically matched controls were examined with sequencing and T-ARMS-PCR for genotyping of *MICA*, and ARMS-PCR for HLA-B*51. The phenotype frequency of MICA*049 (41.5% versus 8.1%, OR = 8.01, *P* = 1.91 × 10^−8^) and HLA-B*51 (46.3% versus 15.7%, OR = 4.62, *P* = 1.21 × 10^−5^) were significantly higher in BD patients than those in controls, whereas MICA*009 showed no significant difference between the two groups (17.1% versus 13.2%, OR = 1.35, *P* = 0.51). After stratification for the effect of HLA-B*51, MICA*049 was still associated with BD in HLA-B*51 negative patients (OR = 40.61, *P* = 0.02). Our results indicate that MICA*049, not MICA*009, is a risk factor to BD, and that is independent from HLA-B*51 in the Han Chinese cohort.

## Introduction

Behçet’s disease (BD) is a multi-system inflammatory disease characterized by recurrent oral and genital ulcers, uveitis, and skin lesions. Although the etiology and pathogenesis of BD are still uncertain, multiple genetic factors have been linked to BD^1,2^. Among them, HLA-B*51 appears to be the most strongly associated known genetic risk to BD in different ethnic groups^2^.

*MICA* (major histocompatibility complex class I chain related gene A), located only 46 kb centromeric of *HLA-B*, is a highly polymorphic gene. It normally expresses on the cell membrane, and functions in immune activation under cellular stress conditions, such as infections, tissue injury, pro-inflammatory signals, and malignant transformation^2^. The *MICA* transmembrane (TM) A6 allele and the MICA*009 allele were associated with BD in multiple previous reports^3–11^. According to updated IMGT/HLA database, there are 107 *MICA* alleles identified. The MICA*009 can be further subtyped into MICA*00901, MICA*0090201 and MICA*0090202. The only difference between the MICA*00901 and the MICA*049 is at codon 335 in exon 6 (https://www.ebi.ac.uk/ipd/imgt/hla/align.html). In the previous studies^5,8–11^, the ambiguity between the MICA*009 allele and the MICA*049 allele was not addressed, because exon 6 was not studied. Therefore, the MICA*009 allele maybe mixed with the MICA*049 allele. Here, we examined the association between *MICA* and BD in a Han Chinese cohort with *MICA* sequencing approach, along with a simple tetra-primer amplification refractory mutation system-polymerase chain reaction (T-ARMS-PCR) method to discriminate between the MICA*00901 allele and the MICA*049 allele.

## Results

The frequencies of *MICA* alleles in the 41 BD patients and 197 healthy controls were shown in Table 1. There were 8 different *MICA* alleles in patients and 16 in controls. The frequency of MICA*049 was significantly higher in the patient group (24.4% in BD versus 4.3% in control, OR = 38.16, *P* = 6.52 × 10^−10^). However, the frequency of MICA*009 (including MICA*009:01 and MICA*009:02) was similar between the two groups (8.5% versus 6.6%, OR = 1.32, *P* = 0.53).

**Table 1:** Comparison of *MICA* alleles between BD patients and controls.

*MICA* Allele	Patients n = 82 (%)	Controls n = 394 (%)	χ^2^	*P* value	OR (95% CI)
002:01	8 (9.8)	65 (16.5)			
004	0 (0.0)	3 (0.8)			
007:01	0 (0.0)	7 (1.8)			
008 (:01 or :04)	13 (15.9)	91 (23.1)			
008:02	0 (0.0)	4 (1.0)			
009:01	7 (8.5)	24 (6.1)	0.39	0.53	1.32 (0.55–3.16)
009:02	0 (0.0)	2 (0.5)			
010:01	22 (26.8)	89 (22.6)			
012:01	3 (3.7)	24 (6.1)			
017	0 (0.0)	5 (1.3)			
018:01	0 (0.0)	1 (0.3)			
019	8 (9.8)	22 (5.6)			
027	0 (0.0)	24 (6.1)			
033	0 (0.0)	1 (0.3)			
045	1 (1.2)	15 (3.8)			
049	20 (24.4)	17 (4.3)	38.16	6.52 × 10^−10^	7.15 (3.55–14.41)

The genotype frequencies of MICA*008(:01 or:04)/MICA*049, MICA*010:01/MICA*049 and MICA*049/MICA*049 were significantly higher in the patients (see Supplementary Table S1).

The *MICA* allele phenotype frequencies in BD patients and controls were shown in Table 2. The MICA*049 was significantly increased in BD patients compared to that in controls (41.5% versus 8.1%, OR = 8.01, *P = *1.91 × 10^−8^). The difference of the MICA*009 frequency between patients and controls was not significant (17.1% in BD versus 13.2% in control, OR = 1.35, *P* = 0.51). The allele frequency of the MICA*A6 was significantly higher in BD patients than that in controls (32.9% versus 11.7%, OR = 3.71, *P* = 1.18 × 10^−6^). The result of phenotype frequency was consistent with that of allele frequency (53.7% versus 21.8%, OR = 4.15, *P* = 3.16 × 10^−5^).

**Table 2:** Phenotype frequencies of *MICA* alleles in BD patients and controls.

*MICA* Allele	Patients n = 41 (%)	Controls n = 197 (%)	χ^2^	*P* value	OR (95% CI)
002:01	8 (19.5)	60 (30.5)			
004	0 (0.0)	3 (1.5)			
007:01	0 (0.0)	5 (2.5)			
008(:01or :04)	11 (26.8)	78 (39.6)			
008:02	0 (0.0)	4 (2.0)			
009:01	7 (17.1)	24 (12.2)	0.43	0.51	1.35 (0.54–3.37)
009:02	0 (0.0)	2 (1.0)			
010:01	20 (48.8)	79 (40.1)			
012:01	3 (7.3)	23 (11.7)			
017	0 (0.0)	4 (2.0)			
018:01	0 (0.0)	1 (0.5)			
019	8 (19.5)	21 (10.7)			
027	0 (0.0)	23 (11.7)			
033	0 (0.0)	1 (0.5)			
045	1 (2.4)	14 (7.1)			
049	17 (41.5)	16 (8.1)	31.59	1.91 × 10^−8^	8.01 (3.58–17.91)

The presence of HLA-B*51 in BD patients and controls were 46.3% and 15.7% (OR = 4.62, *P* = 1.21 × 10^−5^), respectively (Table 3).

**Table 3:** Association of BD with HLA-B*51.

	Patients n = 41 (%)	Controls n = 197 (%)	χ^2^	*P* value	OR (95% CI)
Presence	19 (46.3)	31 (15.7)	19.16	1.21 × 10^−5^	4.62 (2.24–9.54)
Absence	22 (53.7)	166 (84.3)			

To examine whether the observed BD association of MICA*049 and HLA-B*51 are independent from each other, we performed subclonal analysis in HLA-B*51 negative subjects for MICA*049, and in MICA*049 negative subjects for HLA-B*51. As shown in Table 4, the MICA*049 remained significantly associated with BD (OR = 40.61, *P* = 0.02) in HLA-B*51 negative BD patients, but the association of HLA-B*51 with BD appeared lost in MICA*049 negative patients (Table 5).

**Table 4:** Association of MICA*049 with BD stratified for the effect of HLA-B*51.

MICA*049	Absence of HLA-B*51		Presence of HLA-B*51	
	Patients	Controls	Patients	Controls
Presence	2	0	15	16
Absence	20	166	4	15
χ^2^	15.25		3.74	
*P* value	0.02		0.07	
OR (95% CI)	40.61 (1.88–875.58)		3.52 (0.95–13.01)

**Table 5:** Association of HLA-B*51 with BD stratified for the effect of MICA*049.

HLA-B*51	Absence of MICA*049		Presence of MICA*049	
	Patients	Controls	Patients	Controls
Presence	4	15	15	16
Absence	20	166	2	0
χ^2^	1.77		2.00	
*P* value	0.25		0.29	
OR (95% CI)	2.21 (0.67–7.32)		0.19 (0.01–4.23)	

## Discussion

Previously, MICA*009 and MICA*A6 were suggested as susceptibility alleles for BD. The MICA*A6 is a polymorphism with 6 tendent repeats of GCT in exon 5 of *MICA* gene. This polymorphism is included in the MICA*009, and shared by MICA*049 and a number of other *MICA* alleles. In the previous studies^5,8–11^, the *MICA* alleles were identified by PCR-SSP or PCR-SBT based on sequences of exon 2 to exon 5. However, the MICA*00901 and the MICA*049 differ by only one nucleotide at codon 335 of exon 6. Therefore, the ambiguity between these two alleles could not be addressed, and the MICA*009 allele reported in the previous studies may be mixed with MICA*049. According to allelic functional analysis using SIFT program (http://sift.bii.a-star.edu.sg/), the change at codon 335 may impact MICA function.

In the present study, we developed a rapid and cost-efficient T-ARMS-PCR to discriminate the MICA*009 from the MICA*049. Comparison analysis between BD patients and controls showed that the MICA*049, not *009, was strongly associated with BD. As we expected, the MICA*A6 showed a consistent BD association with previous reports as it is within the MICA*049 polymorphism. It is worth noting that the allele frequency of the MICA*009 and *049 in controls were consistent with the previous report of *MICA* alleles in a Chinese population^12^. Considering *MICA* and *HLA-B* genes are located next to each other, and strong linkage disequilibrium (LD) exists between alleles of these two genes, it is necessary to determine whether the observed association is due to LD effect from HLA-B*51. According to the clonal analysis, the MICA*049 was independently associated with BD in the Chinese cohort.

In conclusion, we investigated *MICA* polymorphisms in patients with BD of Chinese Han. It is the first report of MICA*049 in association with BD, and which appeared independent from HLA-B*51. Although the sample size is relatively small in the study, the association achieved significant p value with strong odd ratio. However, it still warrants further validation studies in a larger Chinese cohort and/or other ethnic populations. It may not rule out this observed association is ethnic specific for Chinese Han population.

## Methods

### Participants

A total of 41 Patients (34 male, 7 female) were enrolled between March 2010 and September 2017 from the Eye Hospital of Wenzhou Medical University. The diagnosis of BD was followed the criteria of the International Study Group of BD^13^. The mean age of the patients was 37.8 years (range between 27–50 years) and the mean duration of the disease was 6.4 years (range between 1–18 years). A total of 197 unrelated healthy individuals were recruited in the same geography. All of patients and controls were Chinese Han. The study was approved by the Ethics Committee of the Eye Hospital of Wenzhou Medical University and was conducted according to the Declaration of Helsinki Principles. Written inform consent was obtained from all participants.

### Genomic DNA extraction

Genomic DNA was extracted from peripheral blood cells of all subjects using Bioteke DNA isolation kit (Beijing, China). After detecting DNA concentration by a Nanodrop 2000 spectrophotometer, a part of DNA of each subject was diluted to 10 ng/μl for genotyping assays.

### HLA-B*51 genotyping

For control samples, the HLA-B*51 genotyping was performed with sequence-based typing (SBT) method using secore kits (Life Technologies, USA)^14^. For patients, each sample was genotyped for HLA-B*51 positivity by ARMS PCR method^15^.

### *MICA* genotyping

*MICA* was genotyped by PCR sequencing exon 2–5 regions using bidirectional Sanger sequencing methods^16^. For samples in patient group which were discriminated as MICA*009:01/*049, Sanger sequencing was used to distinguished the two alleles. Two primers (Forward primer: 5′-AGAGAAAGGGCGAATCTGGT-3′, Reverse primer: 5′-AAGAGGGAAA-GTGCTCGTGA-3′) were used to amplify 301 bp PCR products. The PCR was performed in a total volume of 20 μl containing 10 μl of 2 × Taq Master Mix (Jinan, Shanghai, China), 0.4 μM of each primer (Invitrogen, Shanghai, China) and 10 ng of genomic DNA. PCR was carried out on a Veriti Thermal cycler. The PCR thermal cycling condition was an initial denaturation at 94 °C for 3 min, followed by 35 cycles at 94 °C for 20 s, 60 °C for 20 s and 72 °C for 20 s, and a final extension at 72 °C for 5 min. For samples in control group which were detected as MICA*009:01/*049, T-ARMS-PCR was used to differentiate MICA*009:01 from MICA*049. The sequence of the four primers and concentration of each primer were listed in Table 6. Product sizes were 246 bp for T allele, 182 bp for C allele, and 382 bp for the forward outer primer and reverse outer primer. The PCR was performed in a final volume of 10 μl containing 5 μl of 2 × Hot-start Taq Red Master Mix (PHENIX, CA, USA), 0.4–1.4 μM of each primer (IDT, Skokie, USA) and 10 ng of genomic DNA. The PCR program on the Veriti Thermal cycler was as follow: 95 °C for 10 min; 35 cycles of 20 s at 94 °C, 30 s at 62 °C and 25 s at 72 °C, followed by a final extension of 5 min at 72 °C. The results of T-ARMS-PCR were verified by DNA sequencing. Direct sequencing was done with the two outer primers. The PCR products were purified using DNA clean & concentrator kit (Irvine, CA, USA), then the purified PCR products were sent to company for sequencing (GENEWIZ, NJ, USA). The sequencing data were analyzed using chromas software.

**Table 6:** Primers for the T-ARMS-PCR to distinguish MICA*009:01 from MICA*049.

Primer	Sequence (5′ to 3′)	Concentration (μM)
FO	GATGGGAGGGAACTGGTAGGGGCT	0.4
FI	GGTCCTGGATCAACACCCAGTTGGTAC	0.8
RI	GGCATCCCTGTGGTCACGCA	1.4
RO	AGGCACCAAGAGGGAAAGTGCTCG	0.4

FO: Forward outer primer, FI: Forward inner primer, RI: Reverse inner primer, RO: Reverse outer primer.

### Statistical analysis

HLA-B*51 and *MICA* allelic frequencies were calculated by direct counting. The significance of the distribution of alleles between the patient group and the control group was calculated by Chi-square or Fisher’s exact test using SPSS22.0 or Epi info software. If the cell frequency as zero, the odds ratio (OR) was calculated using MedCalc software (https://www.medcalc.org/calc/odds_ratio.php).

## Supplementary information

Table S1 Comparison of MICA alleles in pair between BD patients and controls

## Data Availability

The data generated and/or analyzed in the current study are available from the corresponding authors on reasonable request.
