# Supplementary material for: MICA*049, not MICA*009, is associated with Behçet’s disease in a Chinese population
Source: Sci Rep. 2019 Jul 26;9:10856. doi: 10.1038/s41598-019-47289-z (PMC6659628; doi:10.1038/s41598-019-47289-z)
Supplement: Supplementary file 1 — Table S1 Comparison of MICA alleles in pair between BD patients and controls [file 41598_2019_47289_MOESM1_ESM.docx]

MICA*049, not MICA*009, is associated with Behçet's disease in a Chinese population

Weifeng Zhu^1,2^, Yan Deng^2,3^, Jiucun Wang^4,5,6^, Xinjian Guo^2^,Weifeng Ding^2,7^, Jiashuo Chao^8^, Dan Lin^9^, Yuqin Wang^9^ & Xiaodong Zhou^2^

**^1^**Department of Biochemistry and Molecular Biology, College of Basic Medical Sciences, Nanchang University, Nanchang, China

**^2^**Department of Internal Medicine, McGovern Medical School, University of Texas Health Science Center at Houston, Houston, Texas, USA

**^3^**Department of Ophthalmology of Children, the Second Affiliated Hospital of Nanchang University, Nanchang, China

^4^State Key Laboratory of Genetic Engineering, Collaborative Innovation Center for Genetics and Development, School of Life Sciences, Fudan University, Shanghai, China

^5^ Institute of Rheumatology, Immunology and Allergy, Fudan University, Shanghai, China

^6^ Human Phenome Institute, Fudan University, Shanghai, China

^7^ Department of Laboratory Medicine, Affiliated Hospital of Nantong University, Nantong, China

^8^The First Clinic Medical College, School of Medicine, Nanchang University, Nanchang, China

^9^ Department of Uveitis, the Eye Hospital, Wenzhou Medical University, Wenzhou, China

**Correspondence**

Xiaodong Zhou

Department of Internal Medicine, McGovern Medical School, University of Texas Health Science Center at Houston, Houston, Texas, USA

Email: Xiaodong.Zhou@uth.tmc.edu

Yuqin Wang

Department of Uveitis, the Eye Hospital, Wenzhou Medical University, Wenzhou, China

Email: yqwang57@163.com

Table S1. Comparison of *MICA* alleles in pair between BD patients and controls

| *MICA* allele pair | Patients  n=41 (%) | Controls  n=197 (%) | χ^2^ | *P* value | OR (95% CI) |
| --- | --- | --- | --- | --- | --- |
| *002:01/*002:01 | 0 (0.0) | 5 (2.5) |  |  |  |
| *002:01/*007:01 | 0 (0.0) | 1 (0.5) |  |  |  |
| *002:01/*008(:01or :04) | 1 (2.4) | 14 (0.5) |  |  |  |
| *002:01/*009:01 | 1 (2.4) | 4 (2.0) |  |  |  |
| *002:01/*009:02 | 0 (0.0) | 1 (0.5) |  |  |  |
| *002:01/*010:01 | 2 (4.8) | 21 (10.7) |  |  |  |
| *002:01/*012:01 | 1 (2.4) | 2 (1.0) |  |  |  |
| *002:01/*017 | 0 (0.0) | 1 (0.5) |  |  |  |
| *002:01/*019 | 2 (4.8) | 5 (2.5) |  |  |  |
| *002:01/*027 | 0 (0.0) | 3 (1.5) |  |  |  |
| *002:01/*045 | 1 (2.4) | 3 (1.5) |  |  |  |
| *004/*008(:01or :04) | 0 (0.0) | 1 (0.5) |  |  |  |
| *004/*010:01 | 0 (0.0) | 1 (0.5) |  |  |  |
| *004/*012:01 | 0 (0.0) | 1 (0.5) |  |  |  |
| *007:01/*007:01 | 0 (0.0) | 2 (1.0) |  |  |  |
| *007:01/*012:01 | 0 (0.0) | 2 (1.0) |  |  |  |
| *008(:01or :04)/*008(:01or :04) | 2 (4.8) | 13 (6.6) |  |  |  |
| *008(:01or :04)/*008:02 | 0 (0.0) | 2 (1.0) |  |  |  |
| *008(:01or :04)/*009:01 | 1 (2.4) | 5 (2.5) |  |  |  |
| *008(:01or :04)/*010:01 | 0 (0.0) | 20 (10.2) |  |  |  |
| *008(:01or :04)/*012:01 | 1 (2.4) | 4 (2.0) |  |  |  |
| *008(:01or :04)/*017 | 0 (0.0) | 1 (0.5) |  |  |  |
| *008(:01or :04)/*019 | 2 (4.8) | 5 (2.5) |  |  |  |
| *008(:01or :04)/*027 | 0 (0.0) | 6 (3.1) |  |  |  |
| *008(:01or :04)/*033 | 0 (0.0) | 1 (0.5) |  |  |  |
| *008(:01or :04)/*045 | 0 (0.0) | 3 (1.5) |  |  |  |
| *008(:01or :04)/*049 | 4 (9.8) | 3 (1.5) | 8.06 | 0.02 | 6.99 (1.12-49.09) |
| *008:02/*010:01 | 0 (0.0) | 1 (0.5) |  |  |  |
| *008:02/*012:01 | 0 (0.0) | 1 (0.5) |  |  |  |
| *009:01/*010:01 | 2 (4.8) | 4 (2.0) |  |  |  |
| *009:01/*012:01 | 0 (0.0) | 3 (1.5) |  |  |  |
| *009:01/*018:01 | 0 (0.0) | 1 (0.5) |  |  |  |
| *009:01/*019 | 1 (2.4) | 2 (1.0) |  |  |  |
| *009:01/*027 | 0 (0.0) | 3 (1.5) |  |  |  |
| *009:01/*045 | 0 (0.0) | 1 (0.5) |  |  |  |
| *009:01/*049 | 2 (4.8) | 1 (0.5) |  |  |  |
| *009:02/*049 | 0 (0.0) | 1 (0.5) |  |  |  |
| *010:01/*010:01 | 3 (7.3) | 10 (5.1) |  |  |  |
| *010:01/*012:01 | 1 (2.4) | 5 (2.5) |  |  |  |
| *010:01/*019 | 3 (7.3) | 6 (3.1) |  |  |  |
| *010:01/*027 | 0 (0.0) | 5 (2.5) |  |  |  |
| *010:01/*045 | 0 (0.0) | 3 (1.5) |  |  |  |
| *010:01/*049 | 8 (19.5) | 3 (1.5) | 24.91 | 4.61×10^-5^ | 15.68 (3.47-94.59) |
| *012:01/*012:01 | 0 (0.0) | 1 (0.5) |  |  |  |
| 012:01/*027 | 0 (0.0) | 1 (0.5) |  |  |  |
| 012:01/*045 | 0 (0.0) | 2 (1.0) |  |  |  |
| 012:01/*049 | 0 (0.0) | 1 (0.5) |  |  |  |
| *017/*017 | 0 (0.0) | 1 (0.5) |  |  |  |
| *017/*049 | 0 (0.0) | 1 (0.5) |  |  |  |
| *019/*019 | 0 (0.0) | 1 (0.5) |  |  |  |
| *019/*027 | 0 (0.0) | 1 (0.5) |  |  |  |
| *019/*049 | 0 (0.0) | 1 (0.5) |  |  |  |
| *027/*027 | 0 (0.0) | 1 (0.5) |  |  |  |
| *027/*049 | 0 (0.0) | 3 (1.5) |  |  |  |
| *045/*045 | 0 (0.0) | 1 (0.5) |  |  |  |
| *045/*049 | 0 (0.0) | 1 (0.5) |  |  |  |
| *049/*049 | 3 (7.3) | 1 (0.5) | 9.52 | 0.02 | 15.47 (1.19-816.25) |
